# Early developmental bisphenol-A exposure sex-independently impairs spatial memory by remodeling hippocampal dendritic architecture and synaptic transmission in rats

**DOI:** 10.1038/srep32492

**Published:** 2016-08-31

**Authors:** Zhi-Hua Liu, Jin-Jun Ding, Qian-Qian Yang, Hua-Zeng Song, Xiang-Tao Chen, Yi Xu, Gui-Ran Xiao, Hui-Li Wang

**Affiliations:** 1 College of Food Science and Engineering, Hefei University of Technology, Hefei, Anhui 230009, PR China; 2 School of Pharmacy, Anhui Medical University, Hefei 230038, PR China

## Abstract

Bisphenol-A (BPA, 4, 4′-isopropylidene-2-diphenol), a synthetic xenoestrogen that widely used in the production of polycarbonate plastics, has been reported to impair hippocampal development and function. Our previous study has shown that BPA exposure impairs Sprague-Dawley (SD) male hippocampal dendritic spine outgrowth. In this study, the sex-effect of chronic BPA exposure on spatial memory in SD male and female rats and the related synaptic mechanism were further investigated. We found that chronic BPA exposure impaired spatial memory in both SD male and female rats, suggesting a dysfunction of hippocampus without gender-specific effect. Further investigation indicated that BPA exposure causes significant impairment of dendrite and spine structure, manifested as decreased dendritic complexity, dendritic spine density and percentage of mushroom shaped spines in hippocampal CA1 and dentate gyrus (DG) neurons. Furthermore, a significant reduction in Arc expression was detected upon BPA exposure. Strikingly, BPA exposure significantly increased the mIPSC amplitude without altering the mEPSC amplitude or frequency, accompanied by increased GABA_A_Rβ2/3 on postsynaptic membrane in cultured CA1 neurons. In summary, our study indicated that Arc, together with the increased surface GABA_A_Rβ2/3, contributed to BPA induced spatial memory deficits, providing a novel molecular basis for BPA achieved brain impairment.

Gonadal steroid hormones play pivotal roles in brain development and this influence persists and can even determine behavior patterns throughout life^1^,^2^. Although brain-derived (endogenous) estrogens and androgens remain low level in the brain, they can exert direct and indirect influence on brain functions^3^. BPA, a well-known endocrine disruptor which mimics estrogen effects by binding to estrogen receptors, exists ubiquitously in the environment. As an estrogenic chemical, BPA may exert different effects on male and female rats due to the complicated internal environment, such as different hormone level, hormone type and metabolic rate of BPA. Therefore, BPA has been demonstrated to be associated with alteration in sexual dimorphisms of the central nervous system (CNS) and behavioral impairment in rats^1^.

Emerging evidence provided by behavioral studies has linked BPA exposure with memory deficits, but the mechanism still remains elusive. Our previous work has suggested a link between dendritic spine and spatial memory in SD male rats^4^. The functional neural circuits require elaboration of complex dendritic arbors that integrate multiple synaptic inputs and proper navigation of axons to their targets. Dendritic arborization is of great importance to proper neuronal connectivity and cognitive function. Dendritic spines, small postsynaptic membrane specializations that protrude from the surface of dendrites, have long been considered to provide morphological and structural basis for synaptic plasticity, one of the important neurochemical foundations of learning and memory. Dendritic spine morphology and number are highly dynamic and variable^5^,^6^, which were reported to be correlated with memory formation.

Dendritic development is regulated by a combination of intrinsic programs and extrinsic factors^7^,^8^. Arc (also known as Arg3.1), an activity-regulated cytoskeleton-associated protein which belongs to the immediate early gene family, is highly expressed in dendrites^9^,^10^, post-synaptic density (PSD)^11^,^12^, and nucleus^13^. It has been demonstrated that Arc regulates spine size and the distribution of spine type^14^. Arc blockade impairs long term potentiation (LTP) maintenance and hippocampal-dependent spatial learning^15^. BPA has long been implicated in the impairment of spine formation and cognition, while whether it functions through Arc has not been reported yet.

In consideration of the crucial roles of gonadal steroid hormones in cognition and the endocrine-disrupting property of BPA, it’s necessary to establish how BPA affects cognition in SD male and female rats. In the present study, we performed MWM experiments to assay the sex-impact of BPA on hippocampus-dependent spatial memory in SD rats. Besides, dendritic arborization, spine morphology and Arc expression were analyzed. Further, we examined whether and how BPA affected synaptic transmission in cultured hippocampal CA1 neurons. This study, for the first time, systematically investigated the relationship between BPA induced spatial memory deficits and dendritic development, spine morphology and synaptic transmission, providing novel molecular mechanism for BPA induced cognition deficits.

## Results

### BPA impaired spatial memory in SD male and female rats

**Figure 1:**
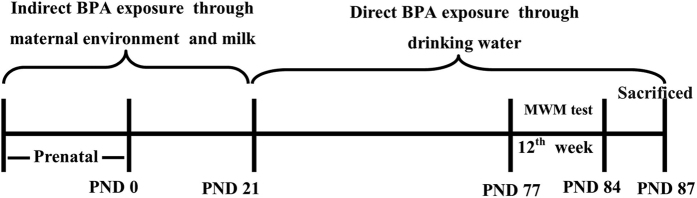
Illustration of the overall research design timeline.

**Figure 2:**
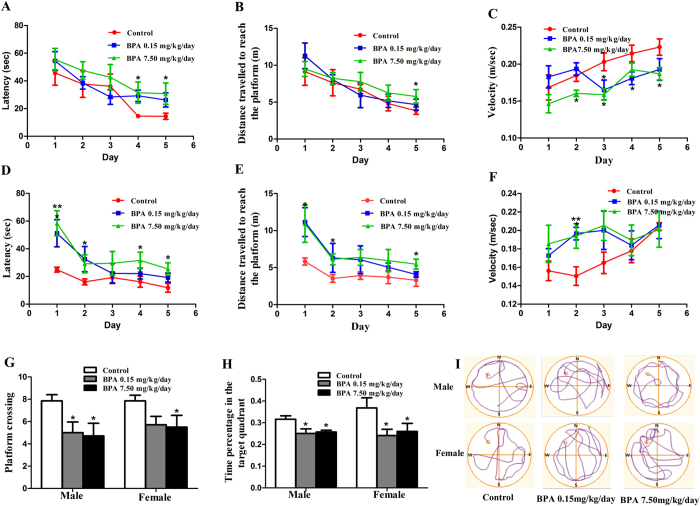
Effects of BPA exposure on SD male and female rats MWM performance. Latency (**A/D**), distance travelled to reach the platform (**B/E**), velocity (**C/F**), platform crossings (**G**) and time percentage in target quadrant (**H**) by male and female rats during MWM training tests, respectively. (**I**) Representative swimming paths of control and BPA exposed rats in the probe test of the MWM experiment. The directions “North”, “South”, “East”, and “West” are indicated as “N”, “S”, “E”, and “W”, respectively. The “North-West” quadrant was the target quadrant (*p < 0.05, **p < 0.01). There were 6, 7, 7 male rats and 6, 7, 6 female rats in Control, BPA 0.15 mg/kg/day and BPA 7.50 mg/kg/day group, respectively.

Morris water maze (MWM) test was employed to assay the effect of BPA on spatial memory in SD rats. The offspring were exposed to BPA as illustrated in Fig. 1. Both male and female rats showed a progressive reduction of the average distance and latency to find the hidden platform during the training period of 5 successive days (Fig. 2A,B,D,E). Meanwhile, probe tests showed that the main factor of BPA treatment significantly affected the time spent in the target quadrant and the number of crossing platform (F_(2, 35)_ = 3.837, p = 0.033; F_(2, 35)_ = 6.561, p = 0.004, respectively). No significant changes were observed following interaction of sex × BPA treatment (F_(2, 33)_ = 0.346, p = 0.711; F_(2, 33)_ = 0.094, p = 0.910, respectively) or main factor of sex (F_(1, 35)_ = 0.888, p = 0.354; F_(1, 35)_ = 0.187, p = 0.669, respectively) (Fig. 2G,H).

These data indicated that both acquisition and retention of spatial memory were impaired upon BPA exposure. Strikingly, we found that BPA induced spatial memory deficits were sex-independent.

### BPA decreased dendritic complexity

**Figure 3:**
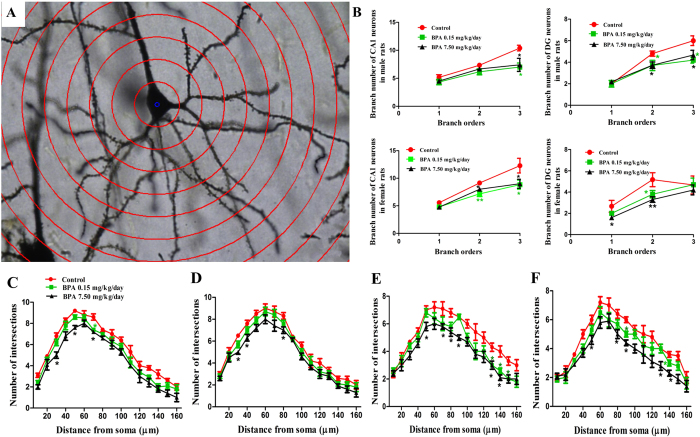
Effects of BPA exposure on dendritic arbor development. (**A**) Representative images of Golgi-Cox impregnated CA1 pyramidal neuron measured by Sholl analysis. (**B**) Quantification of dendrite number at different branch order in CA1 and DG neurons of SD rats. Sholl analysis of the dendritic branch of CA1 (**C/D**) and DG (**E/F**) neurons in SD male and female rats, respectively (*p < 0.05, **p < 0.01). N = 6 or 7 rats and n = 30∼35 neurons per group.

The impaired spatial memory prompted us to explore whether BPA altered neuronal connectivity in hippocampus. As dendritic arborization has been reported to be required for proper neuronal connectivity and network, we then analyzed the dendrite number at different branch order. For male rats, dendrite number decreased significantly only at tertiary branch order (Control, 10.40 ± 0.51; BPA 0.15 mg/kg/day, 7.00 ± 0.73, p < 0.05; BPA 7.50 mg/kg/day, 7.40 ± 1.17, p < 0.05) following BPA exposure in CA1 (Fig. 3B). While in DG, dendrite number decreased dramatically at secondary (Control, 4.80 ± 0.24; BPA 0.15 mg/kg/day, 3.70 ± 0.47, p < 0.05; BPA 7.50 mg/kg/day, 3.72 ± 0.30, p < 0.05) and tertiary branch order (Control, 6.00 ± 0.45; BPA 0.15 mg/kg/day, 4.20 ± 0.29, p < 0.05; BPA 7.50 mg/kg/day, 4.67 ± 0.44, p < 0.05) (Fig. 3B). No obvious changes were observed in the dendrite number at primary branch order in hippocampal CA1 and DG in male rats. For female rats, BPA caused a significant reduction in dendrite number at secondary (Control, 9.14 ± 0.26; BPA 0.15 mg/kg/day, 7.17 ± 0.49, p < 0.01; BPA 7.50 mg/kg/day, 8.00 ± 0.34, p < 0.05) and tertiary branch order in CA1 (Fig. 3B, Control, 12.29 ± 1.33; BPA 0.15 mg/kg/day, 8.83 ± 0.83, p < 0.05; BPA 7.50 mg/kg/day, 9.00 ± 0.82, p < 0.05). Whereas in DG, BPA mainly affected the dendrite number at primary (Fig. 3B, Control, 2.67 ± 0.56; BPA 0.15 mg/kg/day, 2.00 ± 0.19, p > 0.05; BPA 7.50 mg/kg/day, 1.60 ± 0.22, p < 0.05) and secondary branch order (Fig. 3B, Control, 5.17 ± 0.65; BPA 0.15 mg/kg/day, 3.80 ± 0.35, p < 0.01; BPA 7.50 mg/kg/day, 3.30 ± 0.26, p < 0.01).

Sholl analysis showed that BPA exposed rats had less intersections between the dendrites and Sholl circles. Briefly, the decrease of intersection number occurred only within 30∼80 μm from the neuronal soma in CA1 pyramidal neuron as shown in Fig. 3C,D. While in DG granule neuron, intersection number decreased mainly from 80 μm away from neuronal soma (Fig. 3E,F).

These results indicated that BPA exposure negatively affected dendritic formation and development. The decreased dendritic complexity may in turn influence the neuronal connection and the whole network in hippocampus, thus resulting in impaired spatial memory.

### BPA altered dendritic spine density and morphology

**Figure 4:**
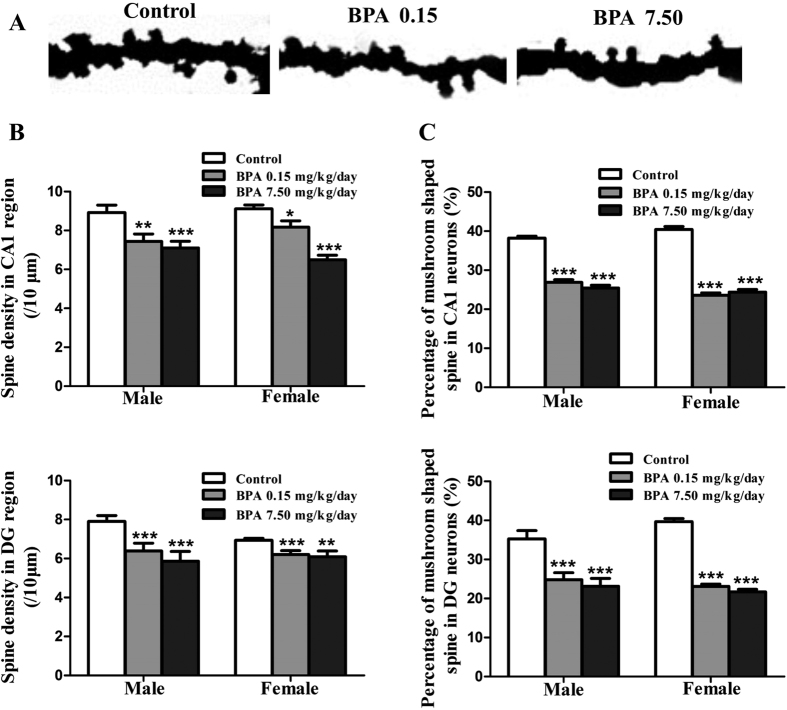
Effects of BPA exposure on dendritic spine density and morphology alteration. (**A**) Representative 10 μm dendritic shaft with spines of hippocampal neurons. The changes of dendritic spine density (/10 μm) (**B**) and the percentage of mushroom shaped spines (**C**) in CA1 pyramidal neuron and DG granule neuron in male and female rats (*p < 0.05, **p < 0.01, ***P < 0.001). N =6  or 7 rats and n = 30∼35 neurons per group.

Further, we examined the alterations of dendritic spine. Dendritic spine density in hippocampal CA1 and DG decreased significantly in a dose-dependent manner, with males exhibiting 15.60% (BPA 0.15 mg/kg/day, p < 0.01) and 19.35% (BPA 7.50 mg/kg/day, p < 0.001) decrease in CA1, respectively. While, females exhibited 9.54% (BPA 0.15 mg/kg/day, p < 0.05) and 28.12% (BPA 7.50 mg/kg/day, p < 0.001) decrease in CA1, respectively (Fig. 4B). Spine density in DG neurons showed a 19.11% (BPA 0.15 mg/kg/day, p < 0.001), 25.70% (BPA 7.50 mg/kg/day, p < 0.001) decrease in male rats and 10.52% (BPA 0.15 mg/kg/day, p < 0.001), 12.25% (BPA 7.50 mg/kg/day, p < 0.01) decrease in female rats, respectively (Fig. 4B).

The mushroom shaped spine is relatively stable and mature among the four types of spines^16^,^17^. We discovered that the percentage of mushroom shaped spines reduced significantly as a result of BPA exposure. Briefly, the percentage of mushroom shaped spines in CA1 neurons decreased by 29.69% and 33.50% in male rats and 41.68%, 39.65% in female rats upon 0.15 mg/kg/day and 7.50 mg/kg/day BPA exposure, respectively (Fig. 4C). In DG, the percentage decreased by 29.66%, 34.36% and 41.81%, 45.39% in male and female rats, respectively (Fig. 4C).

These results suggested that BPA impaired dendritic spine formation and maturation, which may contribute to the impaired spatial memory caused by BPA.

### BPA reduced Arc expression in hippocampus

**Figure 5:**
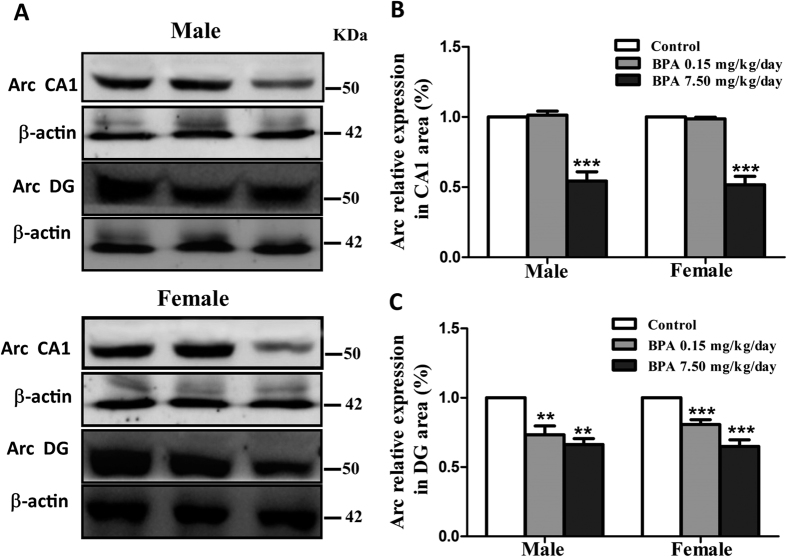
Effects of BPA exposure on Arc expression. Representative immunoblot bands (**A**) and corresponding densitometric analysis (**B/C**) showing Arc protein expression in CA1 and DG region. Blot images were cropped for comparison. β-actin was used as a loading control. Arc and β-actin were run on the same blot. The optical density of bands was quantified by Image-J software. N = 6 or 7 rats per group (**p < 0.01, ***P < 0.001).

The role of Arc in regulating dendrite development and spine formation makes it a potential candidate to explore the mechanism lying behind the dynamic changes of dendritic spine upon BPA exposure. In CA1 region, Arc decreased dramatically only after 7.50 mg/kg/day BPA exposure in both male (Fig. 5B, Control, 1.00 ± 0.00; BPA 0.15 mg/kg/day, 1.00 ± 0.01, p > 0.05; BPA 7.50 mg/kg/day, 0.56 ± 0.06, p < 0.001) and female rats (Fig. 5B, Control, 1.00 ± 0.00; BPA 0.15 mg/kg/day, 1.01 ± 0.03, p > 0.05; BPA 7.50 mg/kg/day, 0.54 ± 0.07, p < 0.001). While, in DG region, Arc decreased significantly upon two doses of BPA exposure (Fig. 5C, male rats, Control, 1.00 ± 0.00; BPA 0.15 mg/kg/day, 0.73 ± 0.06, p < 0.01; BPA 7.50 mg/kg/day, 0.66 ± 0.04, p < 0.01; female rats, Control, 1.00 ± 0.00; BPA 0.15 mg/kg/day, 0.81 ± 0.03, p < 0.001; BPA 7.50 mg/kg/day, 0.65 ± 0.05, p < 0.001). The results indicated that Arc plays a critical role in BPA induced alterations in dendritic spine density and morphology.

### Acute BPA exposure enhanced inhibitory synaptic strength in cultured hippocampal neurons

**Figure 6:**
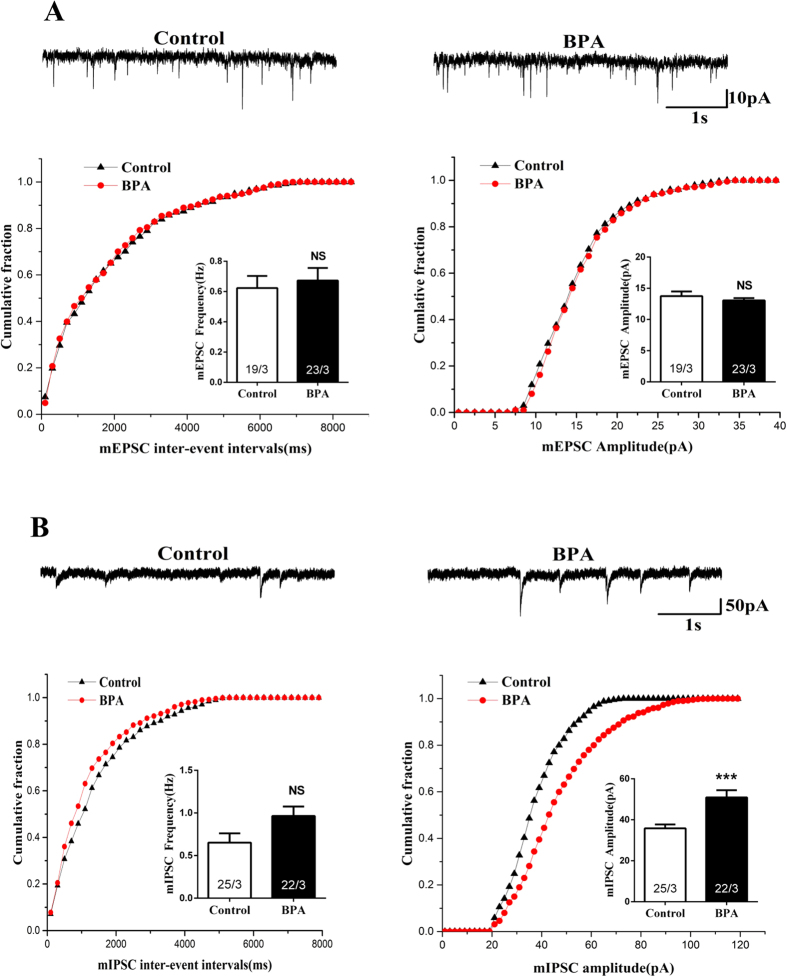
Effects of BPA exposure (2 h) on excitatory and inhibitory synaptic transmission in cultured hippocampal CA1 neurons. (**A**) Representative mEPSC traces and quantification of amplitude and frequency of mEPSC. (**B**) Representative mIPSC traces and quantification of amplitude and frequency of mIPSCs (***p < 0.001). The ‘x’ of ‘x/3’ in the picture represents the number of neurons used from three independent cultures.

Learning and memory are intimately associated with synaptic transmission, we then explored synaptic transmission after acute BPA (2 hrs) exposure. Hippocampal CA1 neurons exposed to BPA showed no significant changes in frequency (Fig. 6A, Control, 0.62 ± 0.08 Hz; BPA, 0.67 ± 0.08 Hz) and amplitude of mEPSC (Fig. 6A, Control, 13.74 ± 0.75 pA; BPA, 13.05 ± 0.38 pA), suggesting that BPA did not affect excitatory synaptic transmission.

Then we wondered whether BPA altered inhibitory synaptic responses. BPA treatment (2 hrs) in cultured hippocampal neurons led to a robust increase in mIPSC amplitude (Fig. 6B, Control, 35.85 ± 1.87 pA; BPA, 50.94 ± 3.48 pA, p < 0.001) without affecting the mIPSC frequency (Fig. 6B, Control, 0.65 ± 0.11 Hz; BPA, 0.96 ± 0.11 Hz), which indicated that acute BPA exposure enhanced inhibitory synaptic transmission.

### BPA recruited GABA_A_ receptor (GABA_A_R) to postsynaptic surface

**Figure 7:**
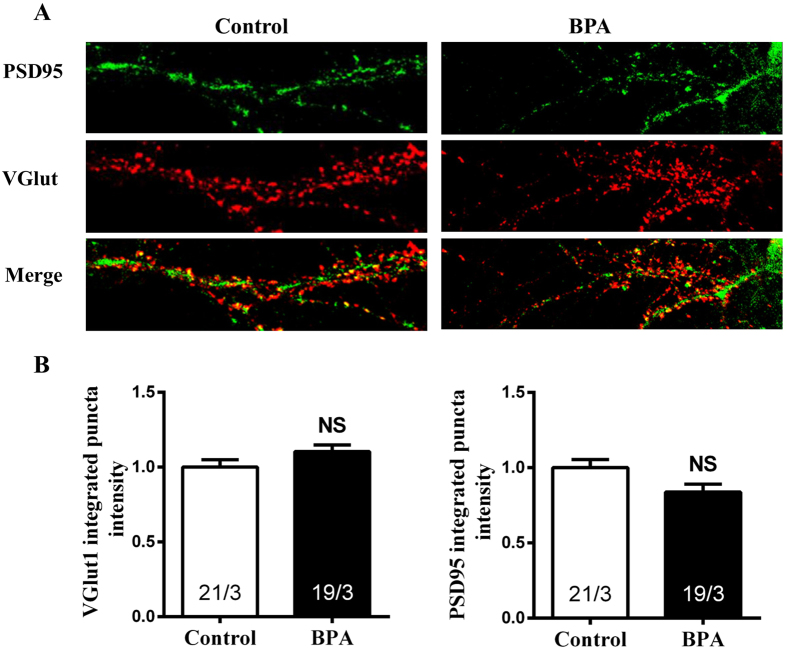
Immunostaining of BPA exposure on excitatory neurotransmission in cultured hippocampal CA1 neurons. (**A**) Immunolabeling of PSD95 (green) and VGlut1 (red). (**B**) Quantification of presynaptic VGlut1 and postsynaptic PSD95 puncta intensity. The ‘x’ of ‘x/3’ in the picture represents the number of neurons used from three independent cultures.

**Figure 8:**
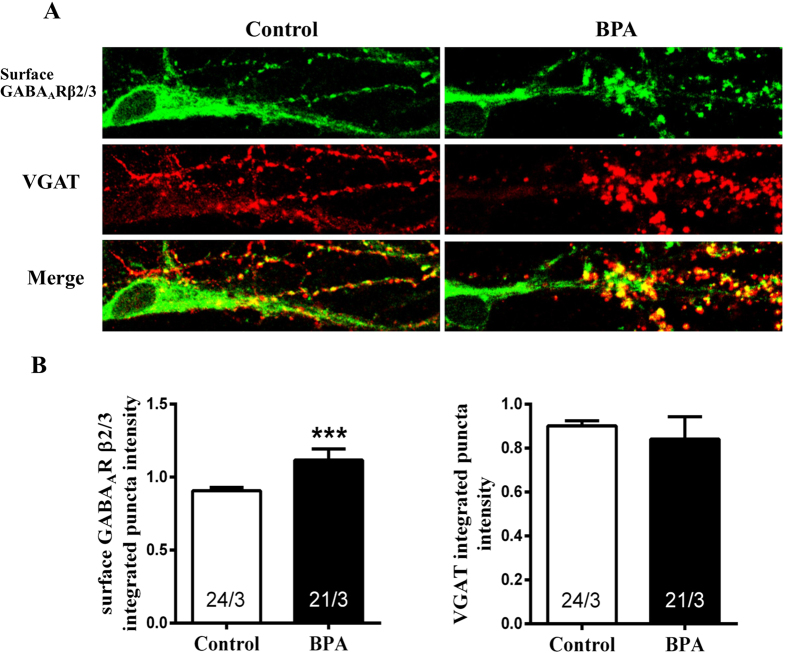
Effects of BPA exposure on postsynaptic surface GABA_A_Rs in cultured hippocampal CA1 neurons. (**A**) Immunolabeling surface GABA_A_Rβ_2/3_ (green) and VGAT (red). (**B**) Quantification of presynaptic VGAT and postsynaptic surface GABA_A_Rβ_2/3_ puncta intensity. The ‘x’ of ‘x/3’ in the picture represents the number of neurons used from three independent cultures (***p < 0.001).

The enhancement of the change in mIPSC amplitude suggested a postsynaptic mechanism. We therefore performed immunocytochemistry experiments measuring the level of synaptic surface-exposed GABA_A_Rs after BPA exposure. We used an antibody to GABA_A_Rβ_2/3_ subunits that recognizes an extracellular epitope to probe surface receptors with vesicular GABA transporter (VGAT) immune-labeling as a general inhibitory presynaptic marker in cultured primary neurons. Consistent with the electrophysiological experiments, integrated puncta intensity of the excitatory synaptic markers, vesicular glutamate transporter 1 (VGlut1), a presynaptic excitatory marker (Fig. 7B, Control, 1.00 ± 0.050; BPA, 1.10 ± 0.04) and PSD95, a postsynaptic excitatory marker (Fig. 7B, Control, 1.00 ± 0.05; BPA, 0.84 ± 0.05), had no significant changes after BPA exposure. While, we found that BPA exposure significantly increased the postsynaptic abundance of GABA_A_R, manifested as increased surface GABA_A_Rβ2/3 integrated puncta intensity (Fig. 8B, Control, 0.91 ± 0.02; BPA, 1.12 ± 0.08) assessed by immunofluorescence experiments. No significant changes were observed in VGAT integrated puncta intensity following BPA exposure when compared with control (Fig. 8B, Control, 0.90 ± 0.02; BPA, 0.84 ± 0.10). Thus, the abundance of surface GABA_A_Rβ2/3 in postsynaptic membrane may, at least in part, account for the increased mIPSC amplitude.

## Discussion

Previous work in our lab has demonstrated that BPA exposure impairs spatial memory and hippocampal dendritic spine formation through Wnt signaling pathway in SD male rats^4^. While, there are still some major questions remaining to be clarified. As a xenoestrogen chemical, whether the effect of BPA on spatial memory is sex-specific? Whether BPA impaired spatial memory by altering the network and neuronal connection in hippocampus? Whether and how BPA influences synaptic transmission?

To address these questions, we performed MWM experiments in SD male and female rats and discovered that the impairment of spatial memory induced by BPA is sex-independent. Furthermore, decreased dendrite number at different branch order and intersections across concentric circles were detected. Then we observed decreased dendritic spine density, altered spine morphology, along with reduced Arc expression in hippocampal CA1 and DG regions. Finally, we discovered that BPA enhanced postsynaptic strength accompanied by increased surface GABA_A_R.

Consistent with the studies of other groups^18^,^19^, we previously discovered that BPA exposure impaired spatial memory in SD male rats. Thus it was rational to ask whether BPA exerts the same influence on SD female rats. In the present study, the MWM results revealed that BPA impaired spatial memory in a sex-independent manner. Actually, the gender’s role involved in BPA’s CNS impairment was highly debatable^20^,^21^. While, the results vary with animal age, BPA exposure time or period, BPA concentrations and exposure methods. Adolescence is an important developmental stage characterized by hormonal changes which induce structural effects on the brain and subsequently behavior. Perinatal BPA exposure impairs spatial memory in both male and female adolescent rats^22^. However, there are reports showing that BPA administration during early development induces sex-specific changes in adult zebrafish social interactions^23^ and long-term BPA exposure leads to sex-specific effects on anxiety-like behavior in adult mice^21^. Therefore, the effects of BPA on animal behaviors are influenced by multiple factors, including animal age. The impairment of dendrite and spine regardless of sex by BPA in our study may account for the impaired sex-independent spatial memory.

It should be noted that, the offspring used in this study were indirectly exposed to BPA from in-utero environment and maternal milk during early development, for detectable levels of BPA have been found in fluids of human and animals, such as urine^24^, blood^25^ and breast milk^26^. It has been reported that high-dose BPA exposure to pregnant mice affects hippocampal neurogenesis and memory retention in the second generation, which have no experience of BPA exposure^27^. On the one hand, the result suggested that BPA exposure during early development have a long-lasting impact. On the other hand, the result indicated a possibility that BPA may affect the offspring memory by modifying their mother’s behavior. Maternal stress during pregnancy has been linked to adverse behavioral and emotional outcomes in their offspring. And this hypothesis has been evidenced by extensive animal and human studies^28^,^29^.

Hippocampus plays critical roles in learning and memory. Both of them require efficient and functional neuronal networks. There is a circuit in hippocampus with input from the Entorhinal Cortex (EC) that forms connections with the DG and output back to the EC through CA1. Damage of any part of this circuit will affect the process of learning and memory^30^,^31^,^32^,^33^. In the present study, dendritic shaft and spine morphology in CA1 and DG regions were investigated, which helped us understand whether and how BPA affects hippocampal input/output and efficiency. Golgi-cox staining experiments revealed that dendritic complexity was impaired upon BPA exposure, manifested as reduced dendrite number at different branch order and decreased dendritic crossings in CA1 and DG regions. This will, to some extent, lead to a dysfunction of hippocampus-EC circuit as stated above, with its direct or indirect consequences verified by impaired spatial memory. Besides, from the aberrantly altered spine morphology, it could also be concluded that BPA lowered the synaptic efficacy within hippocampus. Another reason for BPA induced spatial memory deficits may be that BPA declined neurogenesis in DG^27^,^34^. DG is among a few brain regions currently known to have high rates of neurogenesis in adult rats. These adult-born neurons can influence certain forms of hippocampus-dependent learning and memory formation^35^,^36^.

The regulation of neuronal cytoskeleton is essential for dendrite development and maintenance. In addition, dendritic spines are the most actin-rich structures in the brain^37^,^38^. As an activity-regulated cytoskeleton-associated protein, Arc is an ideal candidate for regulating dendrite and spine. Moreover, global or local deletion of Arc in the hippocampus has been demonstrated to impair the spatial learning consolidation^39^,^40^. Therefore, we quantified Arc expression levels in CA1 and DG after BPA exposure. Interestingly, Arc expression decreased significantly only upon high dose of BPA exposure in CA1 region, but dramatically decreased by both of doses BPA exposure in DG region. The results suggested on the one hand that decreased Arc expression, at least in part, is responsible for BPA induced dendritic branch and spine loss. On the other hand, the results indicated that DG is more sensitive to BPA. The possible explanation of the DG sensitivity is that BPA may negatively affect neurogenesis in DG^34^,^41^ and Arc has been reported to be continually expressed in new-born granule cells^42^. To our knowledge, this is the first report showing that Arc was implicated in BPA induced impaired dendrite and spine formation and spatial memory deficits.

Morphological alterations of dendritic spines have been shown to have profound effects on the efficacy of synaptic transmission^43^. Therefore, we next explored whether the synaptic transmission was affected upon BPA exposure in cultured hippocampal CA1 neurons. The lack of effect on mEPSC frequency and amplitude suggested that BPA may lead to a non-functional spine reduction. However, we found that BPA enhanced inhibitory synaptic strength, accompanied by increased GABA_A_Rs on postsynaptic membrane. Experimental evidence suggests that synaptically released neurotransmitters saturate their receptors^44^ and hence, that the functional strength of GABAergic synapses changes in proportion with the number of postsynaptic GABA_A_Rs^45^,^46^. GABA_A_R activity controls important aspects of brain development and GABA_A_R abnormality is involved in some mental disorders^47^,^48^,^49^. Studies have shown that α4-GABA_A_R knock out (KO) mice exhibited enhanced trace and contextual fear conditioning, along with an enhancement of hippocampus-dependent learning and memory^50^. Inhibition of α5 GABA_A_R attenuated the memory deficits induced by inflammation^51^. Here, we presented that BPA promoted GABA_A_Rβ2/3 clustering to postsynaptic membrane, and the results indicated that the elevated GABA_A_Rβ2/3 in the postsynaptic membrane may be associated with BPA induced spatial memory deficits.

In conclusion, this study investigated the mechanism of BPA induced sex-independent spatial memory deficits. From dendrite development, dendritic spine formation and morphology to synaptic transmission and function, this study systematically elaborated a possible mechanism about how BPA impacted spatial memory. Importantly, our results indicated that Arc may be a potential target of BPA. To our knowledge, this is the first report showing that Arc is involved in BPA induced spatial memory deficits. Our study thus provides unique insights into understanding the molecular basis of BPA toxicity.

## Materials and Method

### Experimental animals

SD rats were supplied by the Laboratory Animal Center, Anhui Medical University, P.R. China. Experiments were performed in accordance with the National Institute of Health Guide for the Care and Use of Laboratory Animals. The study was approved by the institutional animal care and use committee at Hefei University of Technology. SD pups were exposed to BPA indirectly through their mother milk during lactation and then directly after weaning from distilled water containing a series of concentrations of BPA (0 mg/kg/day, 0.15 mg/kg/day, 7.50 mg/kg/day, respectively). The doses mentioned above are much lower than the no-observed-adverse-effect level (NOAEL; 50 mg/kg/day). Animals had free access to food and water. Rats were then subjected to MWM tests.

### MWM experiments

The MWM test was modified from previous studies^52^ and conducted as described previously^4^. In brief, SD rats were subject to MWM tests at 12 weeks of age. The experimental device was a circular tank with a diameter of 160 cm and depth of 70 cm, containing water hold constant at 23 ± 1 °C. SD rats were allowed to swim to the hidden platform with its top surface submerged 1.5 cm below the water level. Each rat performed four trials daily for 5 days. For each trial, the animals were released from a different position in the water maze. The distance travelled to the hidden platform along with latency and velocity were automatically recorded. On the sixth day, the rats were given a 90 seconds retention trial in which the platform was removed. The platform crossings and time spent on the platform quadrant were recorded.

### Western blotting assay

Proteins were extracted as described previously^4^. Briefly, hippocampus was homogenized and dissolved in ice-cold lysis buffer (PBS, pH 7.4) containing a cocktail of protein phosphatase and protease inhibitors (21 μg/ml aprotinin, 0.5 μg/ml leupetin, 4.9 mM MgCl_2_, 1mM sodium-Meta-vanandante, 1% Triton X-100 and 1 mM PMSF) to avoid de-phosphorylation and degradation of proteins. All samples were centrifuged at 14000 × g at 4 °C for 7 min. The supernatant was then assayed for total protein concentration. Proteins were separated in 8.5% SDS-PAGE gel, transferred to PVDF membrane, blocked with 5% non-fat dry milk, followed by incubation with primary antibodies overnight at 4 °C. Then membranes were washed for three times, incubated with secondary antibody. The blots were visualized using ImageQuant LAS 4000 mini system (GE Healthcare). The antibodies of β-actin and Arc were purchased from Abcam (β-actin, 1:2000, ab8227; Arc, 1:1000, ab118929). All results were normalized against β-actin. The analysis was performed using Image J software.

### Golgi-Cox staining assay

SD rats were sacrificed 3 days after the last memory test. The brain was processed by Golgi-Cox staining method as described by Liu *et al*.^4^. In brief, brains were stored in a dark place for two days (37 °C) in Golgi-Cox solution, and then sectioned at 200 μm in 6% sucrose with a vibratome (VT1200, Leica, Germany). All hippocampal sections were collected on 2% gelatin-coated slides. Then slices were stained with ammonia for 60 min, washed with water for three times, followed by Kodak Film Fix for 30 min, and then washed with water, dehydrated, cleared, and mounted using a resinous medium. The neurons in hippocampus were imaged with a Nikon widefield microscope (Eclipse 80i) by using a 40 × objective. Then, we measured spine density and dendritic arbor using concentric circle analysis^53^. The spines counted in the present study were on 2–3 stretches of the secondary dendrite about 10 μm in length. About 6–8 neurons from one animal were selected to analyze the spine morphology and dendrite number. Specially, brains were longitudinally cut into two halves and the left hemisphere was used to examine special proteins expression, while the right hemisphere was processed for morphological staining.

### Primary neuronal cultures

Briefly, hippocampi were dissociated by enzymatic digestion in 0.03% trypsin for 19 min at 37 °C and then triturated with a fire-polished Pasteur pipette. Neurons were plated on poly-L-lysine (0.5 mg/ml; Sigma-Aldrich)-treated 25 mm glass coverslips at a density of 100,000 cells per coverslip for immunocytochemistry, imaging, and electrophysiology experiments. Neurons were exposed to BPA for 2 hrs on 14 day *in vitro* (DIV).

### Immunocytochemistry

Primary hippocampal neurons were fixed with 4% paraformaldehyde in PBS for 10 min and permeabilized with 0.1% Triton X-100 in PBS for 2 min before incubation with primary VGlut1 (Abcam, ab104898, 1:1000), PSD95 (Abcam, ab2723, 1:1000), VGAT (SANTA CRUZ, sc-49574, 1:500) and surface GABA_A_Rβ2/3 (Millipore, MAB341, 1:500) antibodies, followed by incubation of Alexa Fluor-conjugated 488 (FITC) and 568 (CY3) secondary antibodies. Z-stacked images were acquired on Olympus FV1000 BX61WI laser scanning confocal microscope. The integrated puncta intensity of these proteins was analyzed using Matlab software. The experiment was repeated at least 3 times from independent cultures.

### Electrophysiology

The electrophysiological assays were conducted as previously described by Wang *et al*.^54^. Briefly, Whole-cell patch-clamp recordings were performed at room temperature using 14–16 DIV cultured neurons. 4–6 MΩ borosilicate patch pipettes were filled with an internal solution containing (in mM) the following: 120 CsCl, 2 MgCl2, 5 EGTA, 10 HEPES, 0.3Na3-GTP, 4 Na2-ATP, pH 7.35. Cultures were continuously superfused with external solution (in mM) as follows: 100 NaCl, 26 NaHCO3, 2.5 KCl, 11 glucose, 2.5 CaCl2, 1.3 MgSO4 1.0 NaH2PO4). For miniature IPSC (mIPSC) recording, TTX (1 μM), CNQX (10 μM), and APV (50 μM) were included in the perfusion bath. For mEPSC recordings, bath solution contained TTX (1 μM) and picrotoxin (100 μM). Cells were held at −60 mV.

### Statistical analysis

All data were expressed as mean ± SEM. One-way repeated ANOVA was applied to the data that gained from cell culture. Three-way repeated ANOVA was used to assess interaction of sex × treatment × training day during acquisition training in MWM tests. Two-way repeated ANOVA was applied to the data of probe trial in MWM tests, dendrite number, spine alteration and Arc expression. Difference between groups was then tested using Fisher’s protected least significant difference (PLSD) with 95% confidence. A value of p < 0.05 was considered to be statistically significant.

## Additional Information

**How to cite this article**: Liu, Z.-H. *et al*. Early developmental bisphenol-A exposure sex-independently impairs spatial memory by remodeling hippocampal dendritic architecture and synaptic transmission in rats. *Sci. Rep.* **6**, 32492; doi: 10.1038/srep32492 (2016).
